# State Anxiety and Nonlinear Dynamics of Heart Rate Variability in Students

**DOI:** 10.1371/journal.pone.0146131

**Published:** 2016-01-25

**Authors:** Dimitriy A. Dimitriev, Elena V. Saperova, Aleksey D. Dimitriev

**Affiliations:** Department of Biology, Chuvash State Pedagogical University, Chuvash Republic, Russia; University of Washington, UNITED STATES

## Abstract

**Objectives:**

Clinical and experimental research studies have demonstrated that the emotional experience of anxiety impairs heart rate variability (HRV) in humans. The present study investigated whether changes in state anxiety (SA) can also modulate nonlinear dynamics of heart rate.

**Methods:**

A group of 96 students volunteered to participate in the study. For each student, two 5-minute recordings of beat intervals (RR) were performed: one during a rest period and one just before a university examination, which was assumed to be a real-life stressor. Nonlinear analysis of HRV was performed. The Spielberger’s State-Trait Anxiety Inventory was used to assess the level of SA.

**Results:**

Before adjusting for heart rate, a Wilcoxon matched pairs test showed significant decreases in Poincaré plot measures, entropy, largest Lyapunov exponent (LLE), and pointwise correlation dimension (PD2), and an increase in the short-term fractal-like scaling exponent of detrended fluctuation analysis (α1) during the exam session, compared with the rest period. A Pearson analysis indicated significant negative correlations between the dynamics of SA and Poincaré plot axes ratio (SD1/SD2), and between changes in SA and changes in entropy measures. A strong negative correlation was found between the dynamics of SA and LLE. A significant positive correlation was found between the dynamics of SA and α1. The decreases in Poincaré plot measures (SD1, complex correlation measure), entropy measures, and LLE were still significant after adjusting for heart rate. Corrected α1 was increased during the exam session. As before, the dynamics of adjusted LLE was significantly correlated with the dynamics of SA.

**Conclusions:**

The qualitative increase in SA during academic examination was related to the decrease in the complexity and size of the Poincaré plot through a reduction of both the interbeat interval and its variation.

## Introduction

Anxiety is a negative emotional response to threatening circumstances [1]. State anxiety (SA) can be conceptualized as “a state in which an individual is unable to instigate a clear pattern of behavior to remove or alter the event/object/interpretation that is threatening an existing goal” [2]. The neural organization of anxiety spans multiple levels of the brain, from the complex visceral and somatic integration of the limbic system, to the elementary adaptive activity of the brainstem [3]. Anxiety is associated with elevated high blood pressure [4], increased heart rate (HR) [5] and an enhanced respiratory rate [6]. A key system, involved in the generation of this physiological arousal is the autonomic nervous system (ANS) [7]. The ANS responds both to central stimuli and to activation of reflex sensory inputs [8]. The simple reciprocal concept of sympathovagal balance has been the keystone of ANS physiology for many years [9]. Reciprocity is true for many autonomic reflexes, such as the baroreflex [10] or orthostatic stress [11]. In contrast to homeostatic sensory inputs, however, descending influences from rostral brain structures can evoke different patterns of autonomic reactivity, such as reciprocal, independent or coactive changes in the parasympathetic and sympathetic branches of the ANS [7].

The principal property of the ANS is variability. Autonomic outflow has been well established as intrinsically periodic [12, 13]. Some researchers [14, 15] proposed that brainstem autonomic circuits generate this rhythm (the central oscillator theory). This theory is supported by the observation that different oscillations are present in the firing of sympathetic-related neurons of the medulla [16]. The alternative theory (the baroreflex feedback loop theory) postulates that a combination of time delays and feedback results in the oscillation of blood pressure and HR [17, 18]. Mathematical models of the ANS reveal nonlinear properties of these rhythms [19, 20].

Heart rate variability (HRV) is the difference between consecutive instantaneous beat intervals (RR) [21]. HRV may be an independent marker of cardiovascular health [22] and an indicator of ANS activity [23]. The HRV seems to show a beat-to-beat regulation to which the sympathetic and parasympathetic modulatory influences are probably opposite [24, 25]. The physiological background of HRV has been extensively described using statistical and linear spectral analysis methods [26].

A physiological system, that generates the RR time series data, has been conceptualized as a network of biological oscillators with non-linear proprieties [27]. Chaos is apparently a lawless behavior of a nonlinear system totally ruled by deterministic laws [28]. A healthy cardiovascular system is associated with HRV of a chaotic nature; this chaotic nature reflects adaptability, which can be defined as the capacity to respond to unpredictable stimuli [29]. Consequently, nonlinear behavior would indicate greater flexibility and smaller predictability than a linear behavior [30]. Complex temporal patterns of physiological signals can result from interaction between nonlinear oscillatory systems, including those demonstrating chaotic behavior [30].

Different nonlinear measures of HRV quantify different features of nonlinear dynamics of HR. Lyapunov exponents and entropy rates are measures of the dynamics on an attractor. The correlation dimension describes the complex structure of the attractor approximating the fractal dimension. The Poincaré plot describes the evolution of a system. Detrended fluctuation analysis (DFA) quantifies the fractal correlation properties in physiological time series. By combining different nonlinear measures, different aspects of the underlying physiological patterns may be captured [19, 20, 27, 30].

The Poincaré plot is a scatterplot in which current R-R is plotted as a function of previous interval [31]. Poincaré plot analysis is based on a technique from nonlinear dynamics and provides detailed beat-to-beat information on the activity of the sinus node [31]. Analysis of the Poincaré plot can be used to not only to classify the signal into one of various classes (e.g. torpedo, butterfly, parabola, or comet) but also to fit an ellipse, which enables quantification of the Poincaré map [32]. Application of this method includes measurement of autonomic modulation, or randomness, of HR in physiological and clinical studies [32, 33, 34]. Anxiety is associated with a prominent reduction in the standard deviation of the Poincaré plot perpendicular to the line of identity (SD1) [35, 36]. Karmakar et al. [37] proposed a novel descriptor, the Complex Correlation measure (CCM), to quantify the temporal aspect of the Poincaré plot. In contrast to SD1 and dispersion along the line of identity (SD2), this measure incorporates point-to-point variation in the signal.

In time series analysis, time irreversibility refers to the lack of invariance of the statistical properties of a signal under the operation of time reversal [38]. Asymmetric patterns (i.e., those with the ascending side shorter than the descending side or vice versa) suggest irreversibility, but irreversibility might not imply the presence of asymmetrical patterns [39].

Asymmetry is present in physiological systems as it is an essential property of a non-equilibrium system [40]. A visible and statistically highly significant asymmetry has been shown in the Poincaré plot [41]. Porta et al. [39] examined the asymmetry of a Poincaré plot and showed an interrelationship between time irreversibility, pattern asymmetry, and nonlinear dynamics. Recent studies indicate that simple irreversibility indexes are sensitive to autonomic changes during active orthostasis [42] and head-up tilt [39]. Some studies utilized the Poincaré plot in the case of university examinations [43], mental effort [44], and anxiety disorders [35], but the utility of irreversibility indexes and complex correlation measure for anxiety research have not been well defined.

Fishman et al. [45] pioneered an innovative method of temporal Poincaré variability (TPV), which is a novel analysis to quantify the temporal distribution of points and to detect nonlinear sources responsible for physiological variability. Two measures of the Poincaré plot are proposed. The first, called time-delayed TPV (TPVTD) is the measure of the similarity of an interval to its successor. TPVTD is equivalent to SD1; and hence we excluded this method from consideration. The second measure is called long-term TPV (TPVA) and is calculated using the distance from the center of mass to the origin.

Another approach to the nonlinear analysis of HRV is quantification of complexity. The most commonly used non-linear complexity measures are fractal dimensions of various kinds, and measures based on entropy [46].

Entropy is the measure of system randomness and predictability, with greater entropy often associated with more randomness and less system order [46]. The concept of entropy, as it applies to signals such as RR intervals, is to quantify the repetition of patterns in that signal [47]. Pincus [48] developed approximate entropy (ApEn) as a measure of system complexity. ApEn (*m*,*r*,*N)* is approximately the negative natural logarithm of the conditional probability that a dataset of length *N*, having repeated itself within a tolerance *r* for *m* points, will also repeat itself for *m* + 1 points. Reduced ApEn values, indicating large predictability and less complexity in HR dynamics, have been reported in patients with congestive heart failure [49] and schizophrenia [50]. In addition, ApEn increases during exercise have been reported [51]. Cholinergic blockade with atropine does not significantly impact ApEn [52].

To eliminate its limitation of dependency on the record length, Richmann and Moorman modified the ApEn and introduced Sample Entropy (SampEn) [53]. SampEn is precisely the negative natural logarithm of the conditional probability that two sequences similar for m points remain similar for m+1 points, within a tolerance r, excluding self-matches [54]. Thus, a higher value of SampEn also indicates less self-similarity in the time series [54]. Mateo et al. [55] found that pre-competitive SA was associated with low SampEn. However, SampEn has not yet been used as a measure of HRV in studies examining students’ SA.

The hallmark of physiological systems is their extraordinary complexity [56]. Experimental and theoretical evidence suggests that under healthy conditions physiological signals may have a fractal temporal structure [57]. Introduced by Peng and collaborators [58], DFA has become a widely used technique for the determination of (mono-) fractal scaling properties and the detection of long-range correlations in noisy, non-stationary time series. DFA is a scaling analysis method that involves the calculation of a simple quantitative parameter—the scaling exponent α—to represent the correlation properties of a signal. The DFA method may be useful in identifying and quantifying different states of the same system according to its different scaling behaviors. For example, the scaling exponent α for heart interbeat intervals differs between normal and pathological conditions [59]. DFA was originally used to analyze 24-hr Holter recordings [58], but it is impractical for assessing HRV stress responses. Recent studies have reported the susceptibility of short-term HRV to DFA [60]; this was the basis for computation of DFA measures for a 5-min RR sequence. Unmedicated patients with major depressive disorder had a significantly increased DFA when compared with controls [35]. Pre-competitive anxiety is associated with an increased level of the short-term scaling exponent (α1) [54]. However, Mellilo et al. [43] found diminished α1 levels under academic stress.

In recent years, the interest in applying techniques that stem from the chaos theory in studies of electroencephalographic activity [61] and arterial pressure [62] has been increasing. The largest Lyapunov exponent (LLE) is a simple non-linear measure of how fast two initially nearby points on a trajectory will diverge or converge each other in a phase space; LLE quantifies the sensitivity of the system to initial conditions and provides a predictability [63]. As of yet, only a few studies have investigated the impact of acute stressors on HR measures of chaos in healthy individuals. Hagerman et al. [64] demonstrated that in healthy individuals (33–51 years of age), the LLE of HRV significantly decreased during exercise stress. Both chronic and acute stress experiences have been associated with a reduced LLE [43, 65].

In the presence of chaos, the complexity of HR dynamics can be quantified in terms of the properties of the attractor in phase-space, that is, its correlation dimension (D2) [66]. This measure is based on the presumption that dynamics is the output of a deterministic dynamical system, whereas time-domain measures assume that the variability is around a stationary mean and is noise [67]. D2 has been found to be greatly reduced by cholinergic blockade in both animal and human studies [68, 69]. Nahshoni et al. [67] found that patients with major depression had significantly lower mean correlation dimension than healthy subjects. Schubert et al. [65] showed that acute and chronic stresses are both associated with decreases in correlation dimension. The point D2 (PD2) estimate of the correlation dimension was developed by Skinner et al. [70]. Like D2, PD2 describes the complexity of a system (i.e. number of independent variables needed to describe a system). The advantage of PD2 over D2 is its robustness to nonstationarity (i.e., change over the measurement period).

The fact that high HR is associated with lower variability in RR-intervals is well-known [71]. Therefore, it is critical to correct HRV for the prevailing HR, as HR changes significantly in response to academic stress during examination. Sacha and co-workers [72] previously demonstrated that measures of HRV should be corrected by dividing or multiplying with the corresponding mean RR interval.

It is now generally accepted that nonlinear techniques are able to describe HRV in a more effective manner. However, the ability of nonlinear measures of HRV to enhance our understanding of anxiety has only been partly investigated. This paper is focused on the hypothesis that exam stress provokes changes in nonlinear parameters of HRV. Furthermore, we hypothesized that a decrease in HRV is the consequence of a concurrent increase in HR.

## Materials and Methods

The study group consisted of 96 (15 men and 81 women) healthy, nonsmoking volunteers (students of Chuvash State Pedagogical University), whose ages ranged from 19 to 24 years (mean ± SE: 20.53 ± 0.11 years). All the volunteers underwent physical and neurological examinations, as well as routine laboratory tests, lung function test, a 12-channel electrocardiography (ECG) recording, and chest radiographic examination, before the study. No evidence of heart or pulmonary disease was found in any of the subjects. None of the subjects had been taking any medications for at least 2 weeks before the study. On the day of the study, the subjects were instructed to avoid alcohol and caffeinated beverages for the 12 preceding hours and to abstain from heavy physical activity since the day before. The study was approved by the local Ethical Committee for biomedical research of Chuvash State University named I. N. Ulyanov. Written informed consent was obtained from all the volunteers between 19 and 24 years of age (in Russia, the legal age of consent is 18 years).

The mean height and weight of the subjects were 165.25 ± 0.86 cm (range, 145.50–189.50 cm) and 57.06 ± 0.93 kg (range: 41.00–85.00 kg); their body mass index was 20.99 ± 0.28 (range, 16.63–28.58 kg/m2). ECG was recorded in the supine position for 5 min in two different days; the first recording was performed during the controlled resting condition (rest session), while the second one was conducted just before the university verbal examination (exam session). We chose the supine position for physiological and technical reasons. All time series were checked manually by careful visual inspection of the RR intervals, as described previously [73]. Two kinds of methods were used to avoid artifacts, such as false RR detection and ectopic beats. For each record, we first detected artifact and ectopic intervals by using three standard methods, namely percentage filter, standard deviation filter, and median filter [74, 75]. Next, we replaced abnormal RR intervals with the mean value of the neighboring RR intervals that were centered on the ectopic interval. Experiments were conducted at the same time of day (08.00–12.00 h) and in the same room, maintained at 22°C. The Russian version of Spielberger’s State-Trait Anxiety Inventory (STAI), was used to assess SA levels during the rest and exam sessions [76]. The reliability and validity of this version has been evaluated by many researchers [77]. The STAI State Anxiety Subscale evaluates the current state of anxiety by using items that measure subjective feelings of apprehension, tension, nervousness, worry, and associated with arousal of the autonomic nervous system [1]. The students’ STAI scores were classified as low (0–30), moderate (31–45), and high (≥46). Emotional reactivity refers to the tendency to experience frequent and intense emotional arousal. In this study, we examined the intensity of emotional experiences by using the STAI State Anxiety Subscale.

The following variables were used for the non-linear analysis: DFA (with the scaling components α1 and α2), ApEn, SampEn, and the Poincaré plots (SD1, SD2, SD1/SD2, SS, Guzik’s index of asymmetry (GI), and CCM). The Poincaré plots (return maps), correlating the observation n on the x-axis with observation n + 1 on the y-axis, were used to study HRV as a series of discrete events. The primary method for quantifying the Poincaré plot is an ellipse-fitting technique, although the ellipse serves only as a visual guide with no actual mathematical fit of the data to the equation of an ellipse [31]. Brennan et al. [31] developed a method for quantitative assessment of the ellipse, and we used this to estimate SD1, SD2, and SD1/SD2. The shapes of the Poincaré plots being categorized were, according to the value of SD1/SD2: a normal, comet-shaped plot (SD1/SD2 >0.15), and a torpedo-shaped plot (SD1/SD2 <0.15) [32]. We assessed the asymmetry of Poincaré plots by computing GI as follows [41], according to the definitions of clouds proposed by Karmarkar et al. [78] Eq (1):

$$GI=\frac{\sum_{i=1}^{M}{\left(D_{i}\right)}^{2}}{\sum_{i=1}^{N}{\left(D_{i}\right)}^{2}}\times 100\%$$ (1)

where D_i_ is the distance of the plotted points from the line of identity, and M is the number of points in the increasing cloud. The numerator corresponds to the increasing cloud, and the denominator corresponds to the total number of points (N).

A novel extension of the Poincaré plot is the CCM, which measures beat-to-beat dynamics [37]. The CCM was computed in the windowed manner, in which the temporal information of the signal is embedded. The moving window of three consecutive points from the Poincaré plot is considered and the area of the triangle formed by these three points are computed. CCM is composed of all overlapping three-point windows and can be calculated as Eq (2):

$$CCM(m)=\frac{1}{C_{n}(N-2)}\sum_{i=1}^{N-2}\left||A(i)|\right|$$ (2)

where m represents the lag of the Poincaré plot (m = 1), A(i) represents the area of the triangle (formed with i^th^, [i + 1]^th^ and [i + 2]^th^ points of the Poincaré plot) and Cn is the normalizing constant, which is defined as: *C*_*n*_ = *π* × *SD*1 × *SD*2, representing the area of the fitted ellipse over the Poincaré plot.

ApEn (*m*,*r*,*N*) is the negative natural logarithm of the conditional probability that a dataset of length N, having repeated itself within a tolerance r for m points, will also repeat itself for m + 1 points Eq (3). The function is:

$$ApEn=\text{ln}\frac{Am(r)}{Bm(r)}$$ (3)

where *Am*(*r*) is the probability that two sequences will match for *m* points, and *Bm*(*r*) is the probability that two sequences will match for *m* + 1 points [79].

Computation of SampEn is similar to computation of ApEn, with only a small difference, which is SampEn does not count self-matches [54].

Elimination of self-matches makes SampEn more reliable over short data sequences than ApEn [54]. Previous works, in which measures of entropy were calculated for short sequences of RR, have indicated that sample entropy can be accurately estimated from a set of 100 to 5000 data points when the length of the sequences to be compared (m) is set at 1 or 2 and the tolerance level (r) for determining a difference between data points is set between 0.1 and 0.2 of the standard deviation of the total data set [80]. Based on this and other works [81, 82] we choose m = 2 and r = 0.2 × SD.

DFA quantifies the presence or absence of fractal correlation properties of the RR intervals [58]. The DFA procedure [58] consists of four steps. First, the RR series obtained experimentally is integrated by using the expression Eq (4):

$$Y(k)=\sum_{K=1}^{N}\left[RR(i)-RRave\right]$$ (4)

where Y(k) is the k^th^ term of the integrated series (k = 1, 2,…, N); RR(i) is the i^th^ value of the RR intervals, and RRave is the mean of the RR intervals of the original series, with N length. Second, Y(k) is divided into N(t) non-overlapping segments of equal length (t). Next, we detrended the integrated time series, Y(k), by subtracting the local trend, Yn(k), in each box. In step 4 we averaged overall all segments and calculated the square root to obtain the fluctuation function Eq (5) as follows:

$$F(n)=\sqrt{\frac{1}{N}{\sum_{K=1}^{N}\left|Y(k)-Yn(k)\right|}^{2}}$$ (5)

This computation is repeated for all time scales, thereby obtaining a relationship between the mean of the fluctuations [F(n)] and the size of the intervals (n). As F(n) measures the average difference between two interbeat intervals separated by a time lag n, it quantifies the magnitude of the fluctuations over different time scales n [58]. Typically, F(n) will increase with box size n. A linear relationship on a log-log graph indicates a scale exponent law, that is, F(n) ≈ n×α. Under such conditions, the fluctuations can be characterized by a scaling exponent α, which can be calculated by linear regression on a log—log graph [83]. Note that α = 0.5 corresponds to a random walk (a Brownian motion), α = 1 represents 1/f noise and α = 1.5 indicates Brown noise, the integration of white noise [58].

Tan et al. [84] showed that a single exponent is inadequate to describe HR dynamics. Therefore, estimation of short- and long-term exponents, namely α1 and α2, has been proposed to better describe HR dynamics [58]. We calculated α1 to a period of 4 to 11 beats and α2 to periods longer than 11 beats [60].

We estimated LLE by using the algorithm proposed by Rosenstein et al. [85, 86], which has been shown to be particularly useful for small data series. It should be noted that before calculating the LLE, we estimated the number of embedded dimensions and time delay. Delay-time (τ) was determined by using the first minimum of the auto mutual information function. The Cao method was used to estimate the minimal embedded dimension (m) for the present study. Finally, by using τ and m, the phase-space trajectory was reconstructed [87].

Chaotic dimensions were calculated with the pointwise correlation dimension (PD2) algorithm [88] by using the Dataplore^®^ software. TPVA was calculated on a beat-to-beat basis using a software package (http://engineering.case.edu/eecs/research).

In order to remove any mathematical bias from the HRV calculations, we used the HR correction methodology of Sacha et al. [72].

In the present investigation, we sought to address the following question: “Do nonlinear indexes of HRV quantify the autonomic expressions of anxiety, in addition to time- and frequency-domain indexes?” To address this question, we studied SDNN, the time-domain measure of HRV, and LF and HF, the spectral indexes of HRV.

Variables were not normally distributed; therefore, nonparametric statistical methods were applied. The Wilcoxon Matched Pairs Test was first used to detect significant differences in values obtained at different time points (during the rest period and before the examination). The Spearman's rank-order correlation coefficient was calculated to assess the monotonic relationship between variables. Chi-square analysis was used to test proportions. The Mann-Whitney U test was used to analyze differences between the subject groups. Statistical significance was set at p < 0.05. All data were expressed as mean ± SE.

## Results

At rest, all the students had moderate to low STAI scores (mean, 25.98 ± 0.99). Fifty-nine students (61.5%) fell into the low STAI category while 37 (38.5%) had moderate scores. SA was indeed higher in the exam session (41.78 ± 1.05, z = 8.56, p < 0.001). During the exam session, 11 students (11.46%) had low SA scores, 53 (55.21%) had moderate SA scores, and 32 students (33.33%) had high SA scores.

**Fig 1:**
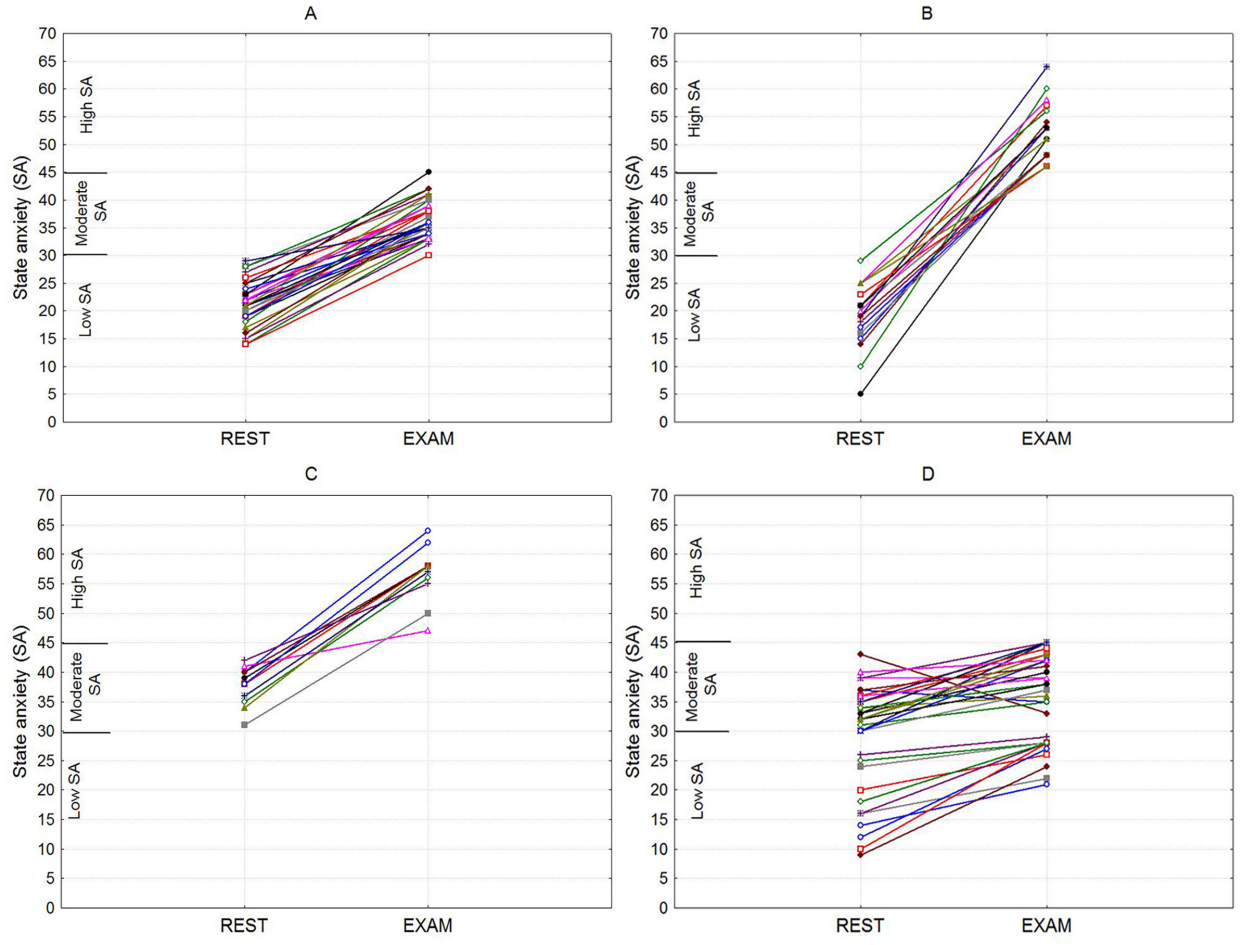
Individual data for examination-induced changes in state anxiety from rest session to the exam session. (A) Increasing from low to moderate anxiety levels. (B) Increasing from low to high anxiety levels. (C) Increasing from moderate to high anxiety levels. (D) Absence of qualitative changes in anxiety.

Individual SA results are summarized graphically in Fig 1.

Most participants (N = 90, 93.7%) showed an increase in SA during the exam session. The transition from the rest session to the exam evoked qualitative increases in SA among the 61 students, from low to moderate in 32 students, from low to high in 18 students, and from moderate to high in 11 students. We divided the participants into two groups based on their anxiety patterns as follows: first, with qualitative increases in state anxiety (N = 61) and second, with no increases (N = 35). The comparison between the two conditions, for the study of nonlinear parameters of HRV, is shown in Table 1.

**Table 1:** Comparison between the two conditions of the study for heart rate variability analysis.

*HRV indexes*	*All participants*		*1-st group*		*2-nd group*	
	Rest	Exam	Rest	Exam	Rest	Exam
**HR [bpm]** (bpm)	72.22±0.93	83.39±1.16#	71.67±1.08	83.62±1.47#	73.36±1.78	82.92±1.87#
**SDNN [ms]**	53.55±1.80	44.78±1.56#	53.75±2.14	45.10±1.77#	53.14±3.32	44.12±3.13#
**LF [ms**^**2**^**]**	800.16±64.15	650.33±51.45*	809.86±76.34	640.96±55.74*	780.25±118.08	669.26±108.76
**HF [ms**^**2**^**]**	1221.42±109.40	673.73±71.60#	1178.45±116.33	607.92±78.66#	1309.62±235.45	808.82±145.58#
**SD1**	35.84±1.54	25.58±1.39#	36.38±1.78	24.57±1.62#	34.73±2.98	27.66±2.62#
**SD2**	65.74±2.12	59.18±1.96#	66.43±2.6	58.75±2.12*	64.32±3.7	60.05±4.16
**SD1/SD2**	0.55±0.02	0.42±0.01#	0.55±0.02	0.4±0.02#	0.54±0.03	0.45±0.02#
**GI**	0.51±0.01	0.48±0.01*	0.51±0.01	0.49±0.01*	0.50±0.02	0.46±0.02
**CCM**	0.26±0.01	0.19±0.01#	0.27±0.01	0.18±0.01#	0.25±0.02	0.21±0.01*
**TPVA**	54.89±1.7	56.44±1.65	54.98±2.1	56.29±1.63	54.71±2.84	56.75±3.83
**ApEn**	1.21±0.01	1.19±0.01	1.22±0.01	1.18±0.02*	1.19±0.02	1.20±0.02
**SampEn**	1.87±0.02	1.68±0.03#	1.89±0.02	1.65±0.04#	1.82±0.04	1.75±0.04
**α1**	0.90±0.02	1.10±0.02#	0.90±0.03	1.13±0.03#	0.89±0.04	1.04±0.04
**α2**	0.83±0.02	0.88±0.02*	0.85±0.02	0.89±0.02	0.80±0.03	0.87±0.03
**LLE**	0.30±0.01	0.25±0.01#	0.31±0.02	0.23±0.01#	0.27±0.02	0.29±0.02
**PD2**	3.75±0.09	3.57±0.07#	3.62±0.10	3.41±0.08#	4.02±0.18	3.90±0.15

Exam vs rest:

* p<0.05;

^#^p<0.01.

Analysis of HR changes during the exam session yielded an overall increase in HR. The change in HR time from baseline did not differ between groups (first group, +11.95 ± 1.51 bpm; second group, +9.57 ± 1.9 bpm; p > 0.05). In our subjects, SDNN and HF were significantly higher during the rest period than during the exam session. The low-frequency component of RR variability decreased significantly during the exam session in the first group. SD1 was significantly lower during the exam session than the rest session in both groups. SD2 decreased significantly before the examination in the first group but not in the second group. Increasing anxiety led to a significant decrease in SD1/SD2 value.

**Fig 2:**
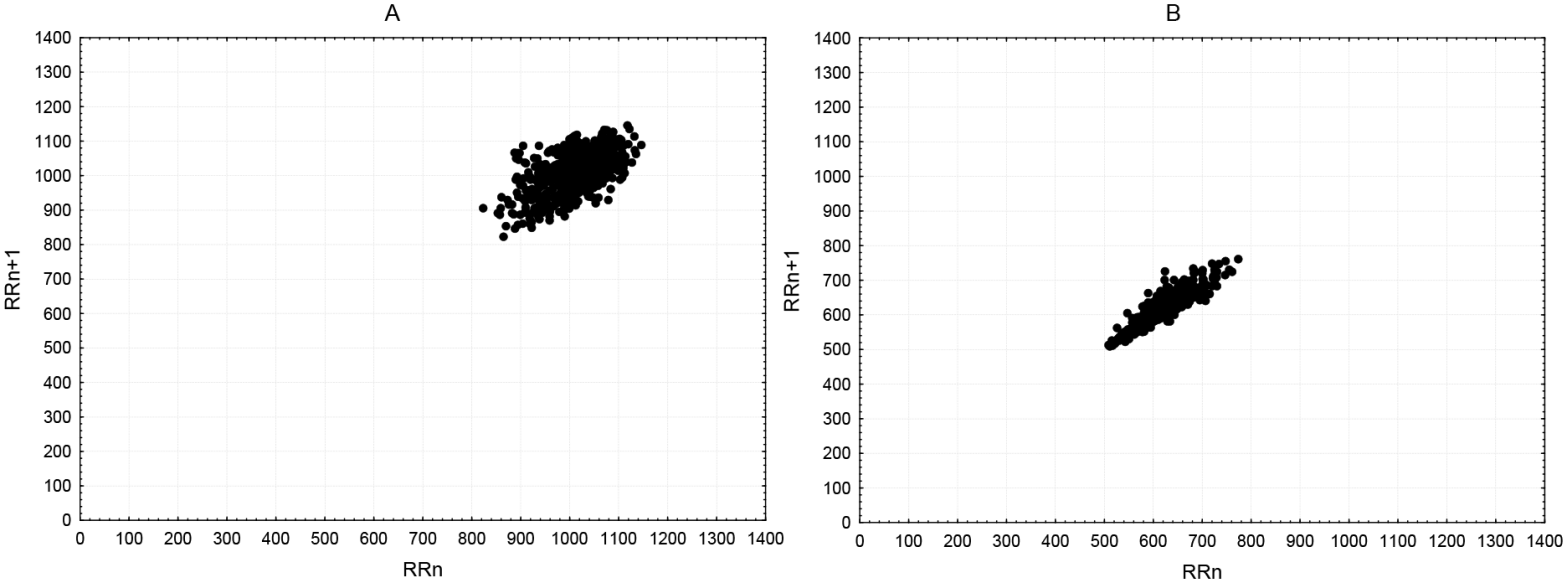
Poincaré plots during the rest (A) and exam sessions (B).

The Poincaré plot at rest displayed a greater dispersion of points than the exam session. Fig 2 shows a significant reduction in area from low SA (rest session) to high SA (exam session), with a contraction of length and a shortening of width in the plots. Compared with a student with low anxiety, the center of the ellipse from a participant with high anxiety is shifted down and to the left. Although the increase in SA induced a significant reduction in the width of the Poincaré plots (Table 1), the correlation between the changes in SD1 and SA did not reach statistical significance (r = –0.08, p > 0.05). Our results did not show a relationship between SD2 dynamics and fluctuations of state anxiety (r = 0.05, p > 0.05). No significant (p > 0.05) differences in the changes in the quantitative measures of Poincaré plot shape were observed between the groups.

Statistical analysis of the ratio between width and length of Poincaré plots (SD1/SD2) revealed a significant decrease in this parameter in both groups. Increased SA was associated with decreased SD1/SD2 (r = –0.20, p < 0.05).

**Fig 3:**
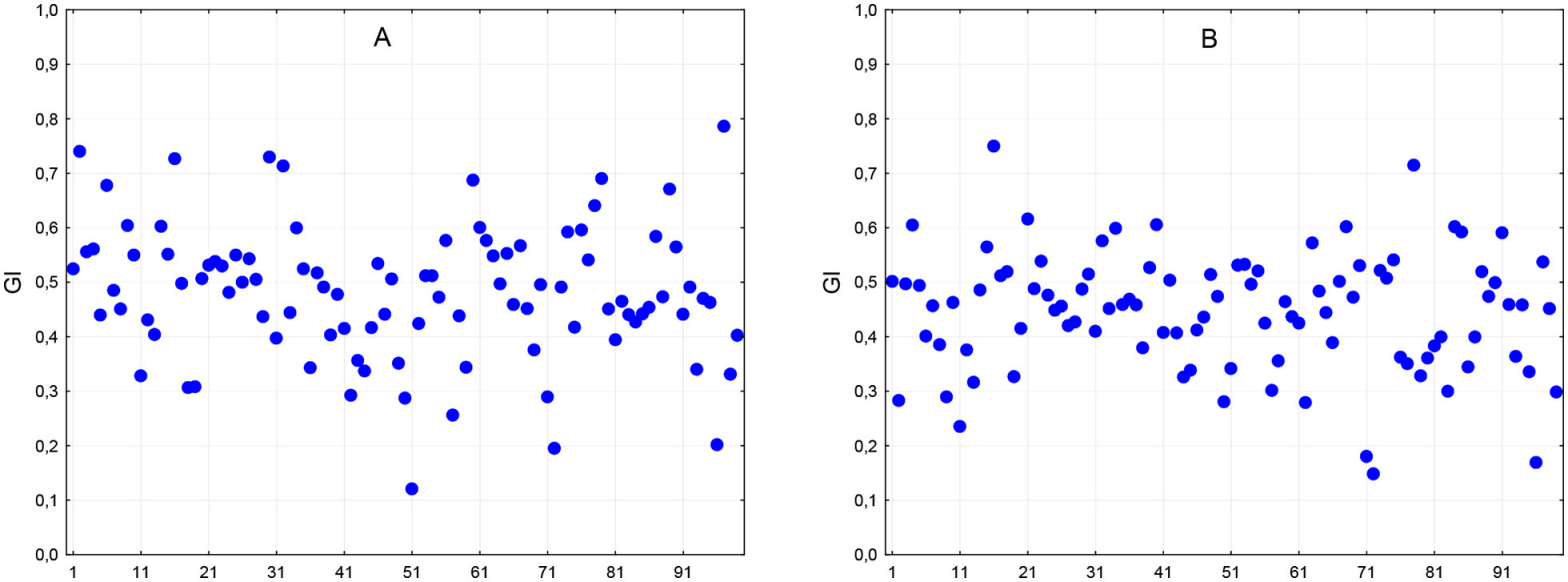
Guzik’s indexes for the rest session (panel A) and exam session (panel B).

In this study, we used the GI range 0.49 to 0.51 as symmetrical [37]. Fig 3 shows the GIs for the rest and exam sessions.

During the rest and exam sessions, 93% and 85% of the subjects, respectively, were found to be asymmetrical. The chi-square analysis demonstrated a non-significant difference between the two sessions (p > 0.05). GIs, measured before examination, were slightly lower, but the difference did not reach statistical significance in either group. The results of the statistical analysis of GI showed no significant difference in response magnitude between the groups (mean GI change: –0.03 ± 0.01 in the first group vs. versus –0.04 ± 0.02 in the second group; p > 0.05). In the assessment of whole range of SA from low to high levels measured at the rest and exam sessions, changes in GI did not show a consistent correlation with the dynamics of SA (r = –0.04, p > 0.05). CCM decreased significantly in both groups, but the changes were greater in the first group (–0.09 ± 0.01 vs.–0.04 ± 0.02; p < 0.05). This decrease was correlated with an increase in SA scores (r = –0.21, p < 0.05). The increment in TPVA between the rest and exam sessions was insignificant, and increases in SA scores showed a weak positive association with increases in TPVA (r = 0.12; p > 0.05).

Increases in SA scores were associated with significant changes in ApEn value in the group of students with high emotional reactivity (Table 1), and the dynamics of ApEn significantly correlated with changes in SA (r = –0.29, p < 0.05). The changes in ApEn score differed between the students who differed in SA scores (mean ApEn change: –0.05 ± 0.02 in first group, vs. 0.014 ± 0.03 in second group; p < 0.05). As shown in Table 1, the mean SampEn values tended to decrease before the exam session in the first group. By contrast, we found that this decrease was significantly less prominent in the second group (–0.07 ± 0.05 vs. –0.25 ± 0.04 in first group; p < 0.01). The SampEn changes correlated significantly with changes in SA scores (r = –0.26, p < 0.05).

The effect of the quantitative increase in SA scores on short—term scaling exponent α1 was significant. The difference in magnitude of the α1 change between the groups was also significant (mean α1 change –0.05 ± 0.02 in first group, vs. 0.014 ± 0.03; p < 0.05).

Our results show a positive correlation between increased SA scores and changes in α1 (r = 0.22, p < 0.05). No association was found between changes in SA scores and α2 (r = 0.1, p > 0.05).

**Fig 4:**
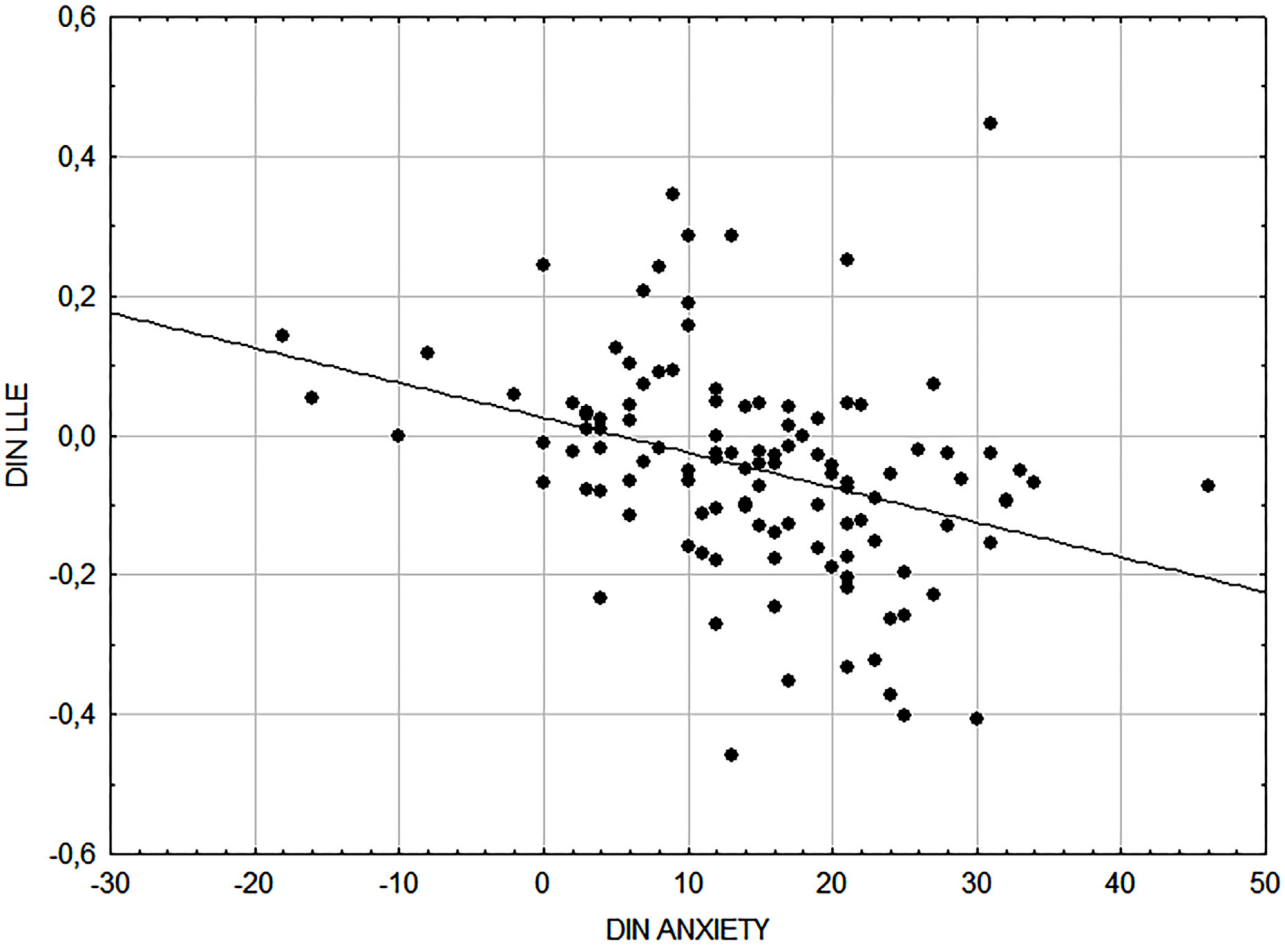
Correlation between the dynamics of state anxiety (DYN SA = SA at exam − SA at rest) and Largest Lyapunov exponent (LLE; DIN LLE = LLE at exam − LLE at rest).

We found significantly lower levels of LLE during the exam session. The changes in LLE differed between the groups (p < 0.01), with negative values found in the first group (mean LLE change –0.08 ± 0.02 in the first group, vs. 0.013 ± 0.02 in second group). By plotting the dynamics of LLE as a function of the corresponding SA scores (Fig 4), we showed that LLE was significantly negatively correlated with SA (r = –0.45; p < 0.05).

In the second group of students, who did not have a qualitative change in anxiety, PD2 did not differ between the sessions (Table 1). However, PD2 was significantly decreased before the examination in the first group. The Pearson analysis demonstrated weak negative correlations between the changes in PD2 and the changes in SA (r = −0.14; p > 0.05).

We examined the correlations between measures of HRV and HR (Table 2).

**Table 2:** Pearson correlations between Poincaré plot dimensions, measures of entropy, short- and long-term exponents, Largest Lyapunov exponent, pointwise correlation dimension, and heart rate.

HRV indexes	Correlation with HR at rest		Correlation with HRat exam		Difference between correlation coefficients (p)
	r	p	r	p	
**SDNN**	-0.470	<0.001	-0.550	<0.001	0.416
**LF**	-0.361	<0.001	-0.499	<0.001	0.203
**HF**	-0.514	<0.001	-0.583	<0.001	0.458
**SD1**	-0.611	<0.001	-0.657	<0.001	0.563
**SD2**	-0.396	<0.001	-0.452	<0.001	0.608
**SD1/SD2**	-0.547	<0.001	-0.636	<0.001	0.303
**GI**	-0.467	<0.001	-0.263	0.004	0.076
**CCM**	-0.551	<0.001	-0.658	<0.001	0.204
**TPVA**	-0.044	0.637	-0.017	0.295	0.839
**ApEn**	0.416	<0.001	-0.016	0.857	0.001
**SampEn**	-0.524	<0.001	-0.599	<0.001	0.410
**alpha1**	0.487	<0.001	0.481	<0.001	0.953
**alpha2**	0.146	0.118	0.329	<0.001	0.151
**LLE**	0.202	0.029	-0.021	0.826	0.091
**PD2**	-0.001	0.993	-0.187	0.044	0.158

HR was significantly negatively correlated with the width (SD1) and length (SD2) of the long and short axes of the Poincaré plot images. The correlations for the exam session were slightly higher than the correlations for the rest session. Correlations between the Poincaré plot axes ratio (SD1/SD2) and HR indicated a strong, negative relationship between these two measures. The asymmetry index GI had a significant negative association with the ratio of HR. HR was negatively correlated with temporal dynamics of the Poincaré plot, estimated by the complex correlation measure (CCM). Although Pearson correlation analysis showed that HR was significantly and negatively associated with all of the above-mentioned indicators of the Poincaré plot, the measure of long-term temporal Poincaré variability TPVA was not significantly correlated with HR. Approximate entropy showed a strong positive association with HR measured during the rest session, but did not have a significant correlation with HR measured before the exam session. Significant negative correlations were observed between HR and SampEn during both sessions, as shown in Table 2. We found that elevated HR was significantly associated with increases in the short-term scaling exponent α1. The long-term scaling exponent α2 showed a statistically significant positive correlation with HR during the exam session. The correlation between PD2 and HR was significant at rest (p < 0.05), based on the Pearson correlation statistical analysis. Baseline levels of LLE were significantly correlated with HR. The results from the present study suggest that the effects of exam stress are partly mediated by increased HR. All conventional linear measures of HRV (SDNN, LF and HF) correlated significantly (P < 0.001) with HR. A statistically significant difference in ApEn was found between the correlation coefficients.

The formulas for correcting HRV are given in S1 Table. Table 3 shows nonlinear HRV indexes, adjusted for HR.

**Table 3:** Heart rate-corrected measures of heart rate variability.

*HRV indexes*	*All participants*		*1-st group*		*2-nd group*	
	Rest	Exam	Rest	Exam	Rest	Exam
**SDNN**	21.67±0.69	21.92±0.60	21.54±0.84	22.18±0.65	21.94±1.22	21.39±1.25
**LF**	1094.85±84.49	1108.66±75.70	1100.89±102.82	1092.98±77.79	1082.45±155.49	1140.85±168.74
**HF**	2243.60±220.78	1863.35±160.08	2214.95±268.06	1608.66±125.57	2302.39±394.17	2386.13±405.93
**SD1**	48.32±1.59	43.94±1.74#	48.74±2.02	42.11±1.92#	47.45±2.53	47.69±3.5
**SD2**	77.54±2.4	79.39±2.39	77.88±3.02	78.91±2.37	76.85±3.99	80.36±5.49
**SD1/SD2**	0.22±0.006	0.20±0.006#	0.22±0.006	0.20±0.008#	0.22±0.01	0.22±0.006
**GI**	17.48±0.23	17.76±0.28	17.54±0.27	18.14±0.29	17.37±0.44	16.96±0.62
**CCM**	0.10±0.003	0.09±0.002#	0.11±0.003	0.09±0.003#	0.10±0.005	0.10±0.003
**ApEn**	1.70±0.012	1.65±0.02	1.71±0.01	1.64±0.02#	1.66±0.03	1.67±0.03
**SampEn**	0.64±0.006	0.62±0.009*	0.65±0.007	0.61±0.011#	0.63±0.012	0.64±0.011
**α1**	0.74±0.017	0.80±0.016#	0.76±0.02	0.82±0.02*	0.73±0.03	0.76±0.03
**α2**	0.24±0.005	0.24±0.004	0.25±0.006	0.24±0.005	0.23±0.009	0.24±0.008
**LLE**	0.86±0.034	0.68±0.031#	0.90±0.04	0.64±0.04#	0.79±0.05	0.78±0.05
**PD2**	0.70±0.02	0.70±0.01	0.67±0.02	0.66±0.015	0.75±0.03	0.075±0.03

Exam vs. rest:

* p<0.05;

^#^p<0.01.

Table 3 presents the adjusted results of the SDNN, LF, HF and nonlinear HRV indexes for the resting and examination conditions. The Wilcoxon test for HR-corrected convenient HRV indexes showed no significant difference between the rest and exam sessions. After correction for HR, the corrected SD1 and SD1/SD2 were still decreased (p < 0.01), while SD2 was slightly increased (p > 0.05). The finding of decreased CCM in the second group (subjects with less pronounced emotional response) during the transition from the rest session to the exam session largely disappeared after adjustment for HR. The results of our analysis show that when HR is taken into account, the difference between the average GI during the rest and exam sessions was decreased to a non-significant level in the first group. HR correction did not influence the change in ApEn, SampEn, and LLE. The use of HR correction formulas led to a significant reduction in PD2 dynamics in both groups. After adjusting for HR, the dynamics of LLE was still significantly negatively associated with the dynamics of anxiety (r = −0.43; p < 0.05). Differences in the changes in the HRV nonlinear parameters (with the exception of DFA) between the groups were not altered after correction for HR.

## Discussion

This study investigated how SA in academic conditions is related to the nonlinear dynamics of HRV. Nonlinear analysis methods are designed to assess the quality, scaling, and correlative properties of signals [29]. We confirmed the effect of increased anxiety on the HR fluctuation by comparing nonlinear HRV indexes between rest and exam sessions. The physiological meaning of Poincaré plot shape and indexes has been examined in different functional states [32, 89, 90]. The relationship between autonomic function and the shape of the Poincaré plot has been established; that is, the narrower the observed pattern, the larger the shift in sympathovagal balance toward an increase in sympathetic nervous system activity [91, 92]. Norepinephrine infusion in healthy volunteers has been shown to cause a sudden change in fixed RR interval dynamics, resulting in a torpedo-shaped Poincaré plot [32]. Meanwhile, atropine administration results in a reduction in the width of the Poincaré cloud [78]. SA is associated with a prominent change in sympathovagal balance [93]; nevertheless, normal comet-shaped scatter plots (and no torpedo-shaped plots) were observed for all subjects in both conditions.

Infusion of atropine induced a reduction in SD1 [87, 94], indicating that the “width” of the Poincaré plot is a measure of parasympathetic nervous system activity [95–97]. Our findings suggest that SA has a predominantly inhibitory effect on parasympathetic activity in exam situations, as the SD1 parameter is significantly lower in exam situations than in the rest situations. SD2 is a nonlinear index with uncertain physiological meaning and interpretation. It is thought to reflect the continuous long-term variability of the RR intervals [89, 98]. Guzik et al. [99] interpreted the SD1/SD2 ratio as a measure of the balance between short- and long-term HRV. During the exam session, the SD1/SD2 ratio decreased significantly owing to the important reduction in SD1 compared with SD2. This highlights the parasympathetic withdrawal and sympathetic activation associated with the transition to a higher level of SA. Our results confirm the effect of anxiety and mental effort on the Poincaré plot [35, 44]. However, our research lends additional insight into the study of SA by using quantitative measures of Poincaré plot shape. In contrast to a previous work [43], we found a significant reduction in the SD2 measured before the examination.

Asymmetry of a Poincaré plot is associated with time irreversibility and nonlinear dynamics [39, 42]. Temporal irreversibility and asymmetry are prominent features of HRV, and differences between HR accelerations and decelerations are related to the physiological or pathological states of organisms [39, 42]. This property was confirmed by analysis of our scatterplots. Our results exhibit prominent asymmetry of the Poincaré plot, indicating that the heart period variability in most of the students was irreversible, regardless of SA levels. Considering that the detection of time irreversibility implies the presence of nonlinear dynamics, we can conclude that short-term heart period variability is nonlinear in a major portion of participants during both sessions [42]. Asymmetry in the GI slightly and non-significantly decreased during the exam session. According to Porta et al. [39], an important shift in sympathovagal balance toward vagal withdrawal is associated with an increase in the asymmetry of Poincaré plot. Tonhajzerova et al. [100] found prominent reductions in the resting HR time irreversibility indexes in adolescent female patients with major depressive disorder. The absence of the effect in our study may have to do with the difference between SA and depression [101].

From the theoretical definition of CCM, it is obvious that this measure quantifies variability in the temporal structure of Poincaré plots [37]. CCM quantifies underlying temporal dynamics in a Poincaré plot; the decrease in CCM indicates increased regularity and decreased variability [37]. The value of CCM decreased with the decrease in parasympathetic activity during atropine infusion and 70° head-up tilt phase test [78]. This suggests that the low CCM during the exam session was caused by an increase in sympathovagal balance. The decrease in CCM indicates a reduction in RR variability and increasing regularity, associated with potential risk of cardiovascular events [102]. Our results can be compared with those from the work of Jellinek et al. [103]. Their study demonstrated a significant reduction in CCM in patients with depression, and they suggest that CCM is more sensitive to parasympathetic nervous system activity, than SD1 and SD2. The increment in TPVA between the rest and exam sessions was insignificant, and the increases in SA level showed a weak positive association with the increases in TPVA (r = 0.12; p >0.05). The authors of the temporal Poincaré variability methodology [45] theorized that this method assesses general patterns of temporal change in HR associated with nonlinear dynamics and complements other time-dependent methods. However, our results do not promote the utility of TPVA as a marker of anxiety-induced changes in HRV.

Entropy, as it relates to dynamical systems, is the rate of information production [46–48], and approximate entropy can be used to classify complex systems, such as physiological systems [53]. Reduction in entropy means greater regularity; this condition is associated with sickness and aging [47]. Previous studies reported discrepant results concerning the effect of negative emotions and stress on complexity measures based on entropy. Valenza et al. [104] found that the mean ApEn decreased significantly during arousal elicited by pictures. Mellilo et al. [43] showed decreased ApEn due to university examination. Anishchenko et al. [105] reported, in healthy young subjects, that short-term psychological stress was associated with both decreases and increases in HR complexity (i.e., approximated entropy). The present data show that ApEn and SampEn decrease significantly in the group of students with high emotional reactivity. The decreases in indexes of HR complexity during the exam session reflects a shift of the sympathovagal balance toward sympathetic predominance [80] and a risk of cardiovascular events [106]. These effects of SA on entropy measures are in line with studies that examined the relationship between HR complexity and high-stress musical performance [107] or arithmetic stress [108].

Physiological investigations have shown that the heart and other physiological networks behave most chaotically when they are young and healthy [57, 59]. The chaotic behavior of healthy physiological networks should not be interpreted as transient perturbations produced by a fluctuating environment, but rather as a necessary component of normal functioning [57]. The output of healthy systems offers a type of complex variability associated with long-range, fractal-like correlations [57]. DFA has been suggested to be the most appropriate method to quantify the fractal properties of a time series of RR intervals. In the present study, the increased values of α1 observed during the shift to higher levels of SA revealed a strong positive association between STAI score and short-term correlations in the RR data. Norepinephrine spillover does not induce prominent changes in short-term scaling exponent [81], but tilt is associated with a significant increase in α1 [51]. Results on vagal blockade with atropine suggest that the increase in the short-range exponent during the exam session was due to cardiac parasympathetic withdrawal. The correlation between the dynamics of SA level and α1 was also related to changes in cardiac vagal activity.

Our results confirm the finding from the study of Valenza et al. [104] in which healthy volunteers were subjected to emotional visual elicitation, along with measurement of nonlinear indexes of HRV during the neutral and arousal sessions. Their research demonstrated that LLE decreased significantly during arousal elicitation. Using the PD2 algorithm of Skinner et al. [88], we showed that statistically significant changes in heartbeat PD2 as SA level increases.

Although the association between traditional measures of HRV and HR is well recognized, we have not found any studies concerning the correlation between HR and nonlinear measures of HRV. We demonstrated that all nonlinear indexes of HRV, except TPVA, are associated with HR. Monfredi et al. [109] proposed a biophysical model that explains this phenomenon. They postulated that “HRV is primarily dependent on HR and cannot be used in any simple way to assess autonomic nerve activity to the heart.” However, the decreases in SD1, SD1/SD2, CCM, SampEn and LLE in response to the stress from academic examination was not altered by correction for HR. By contrast, the association between anxiety and linear HRV measures was greatly attenuated by adjustment for HR. These data strongly suggests that even after adjusting for HR, anxiety induces reductions in the complexity of HRV.

Our findings resemble the Neurovisceral Integration Model [110]. Thayer and Friedman proposed a model that relates anxiety to vagal tone, autonomic flexibility, and adaptability [93, 110]. The model postulates that emotions may be characterized as a reaction to an environmental event that facilitates the rapid mobilization of cognitive, behavioral, and autonomic systems toward action. The efficient interaction between these systems allows for maximal organism flexibility in adapting to a changing environment. According to the neurovisceral integration model, flexibility is an important determinant of adaptation to threatening conditions and anxiety is associated with a systemic inflexibility grounded in poor inhibition [93]. Strong emotions, such fear or phobia, can induce loss of complexity and adaptability [93]. The observed decrease in sample entropy may suggest a shift toward simplification of cardiovascular regulation and reduction of flexibility [106]. Relatively low levels of SD1 and SD2 during the exam session indicate a decrease in HRV associated with an increase in SA; reduced HRV is common in a wide range of maladaptive conditions [93].

The present study is not without limitations. One limitation is that we did not complete the descriptive picture of the nonlinear dynamics of heart rate in anxiety through the use of fuzzy measure entropy, the Hurst exponent, and multiscale entropy. Another limitation may be related to the small number of participants who had decreased SA levels during the exam session. However, these study limitations are balanced by strong points. The strength of this research is that we examined cardiac autonomic functions in a younger cohort of healthy participants without anxiety and depression disorders by using a battery of comprehensive Poincaré plot methods, including computation of complex correlation measures and Guzik’s index of asymmetry.

Most of the studies that investigated academic stress, tended to neglect interindividual differences in intraindividual changes in SA under exam stress. To our knowledge, only a handful of studies considered individual differences in anxious arousal. Although we do not claim absolute originality, the present study differs from previous studies in several ways. First, in keeping with the literature that suggests the existence of distinct types of anxiety style [88, 111, 112, 113], we included two groups of students divided according their patterns of anxiety arousal.

Second, compared with previous studies on exam stress [43, 114, 115, 116], we assessed the correlation between changes in HRV indexes and changes in SA scores. Finally, whereas previous studies only demonstrated the effects of stress on unadjusted nonlinear measures of HRV [43, 65, 108], the present study also provides analyses of HR-corrected HRV indicators. In sum, our approach thus signifies an important step toward understanding how stress influences nonlinear dynamics of HRV.

In conclusion, this study shows that SA is associated with alterations in the complexity of HRV. Our results also suggest that the decrease in HRV and the increase in short-term fractal exponent differed among subjects, and that the prominent loss in the complexity of heart rate variability is associated with a qualitative change in state anxiety.

## Supporting Information

S1 Table Formulae to adjust nonlinear HRV indexes for mean RR (avRR). (DOCX)

S2 Table Heart rate variability and anxiety data. (XLSX)
