# Supplementary material for: State Anxiety and Nonlinear Dynamics of Heart Rate Variability in Students
Source: PLoS One. 2016 Jan 25;11(1):e0146131. doi: 10.1371/journal.pone.0146131 (PMC4726749; doi:10.1371/journal.pone.0146131)
Supplement: S1 Table — (DOCX) [file pone.0146131.s001.docx]

**S1 Table. Formulas to adjust linear and nonlinear HRV indexes for mean RR (avRR).**

| Index | Formula |
| --- | --- |
| SDNN | $SDNNcorr=(SDNN/avRR^{1.5})\times10^{4}$ |
| LF | $LFcorr=(LF/avRR^{2})\times10^{6}$ |
| HF | $HFcorr=(HF/avRR^{4})\times10^{12}$ |
| SD1 | $SD1corr=(SD1/avRR^{2})\times10^{6}$ |
| SD2 | $SD2corr=(SD2/avRR)\times10^{3}$ |
| SD1/SD2 | $SD1/SD2corr=[(SD1/SD2)/avRR^{1.5}]\times10^{4}$ |
| GI | $GIcorr=(GI/avRR^{0.5})\times10^{3}$ |
| CCM | $CCMcorr=(CCM/avRR^{1.5})\times10^{4}$ |
| ApEn | $ApEncorr=ApEn\times avRR^{0.5}$ |
| SampEn | $SampEncorr=(SampEn/avRR^{0.5})\times10$ |
| α1 | $\alpha1corr=(\alpha1/avRR^{0.5})/10^{3}$ |
| α2 | $\alpha2corr=(\alpha2\times avRR^{0.5})/10^{2}$ |
| LLE | $LLEcorr=(LLE\times avRR^{0.5})/10$ |
| PD2 | $PD2corr=PD2/avRR^{0.5}$ |
